# Prognostic values of long non-coding RNA MIR22HG for patients with hepatocellular carcinoma after hepatectomy

**DOI:** 10.18632/oncotarget.23110

**Published:** 2017-12-11

**Authors:** Yuan Dong, Weiwei Yan, Shi-Long Zhang, Mu-Zi-He Zhang, Yan-Ping Zhou, Hai-Hui Ling, Meng Ning, Yanling Zhao, Ang Huang, Ping Zhang

**Affiliations:** ^1^ Department of Pharmacy, 302 Hospital of People’s Liberation Army, Beijing, People’s Republic of China; ^2^ Liver Cancer Center, 302 Hospital of People’s Liberation Army, Beijing, People’s Republic of China; ^3^ Non-Infectious Liver Disease Center, 302 Hospital of People’s Liberation Army, Beijing, People’s Republic of China; ^4^ Chinese Medicine Pharmacy of Integrative Medicine Center, 302 Hospital of People’s Liberation Army, Beijing, People’s Republic of China

**Keywords:** MIR22HG, prognosis, hepatocellular carcinoma

## Abstract

Hepatocellular carcinoma (HCC) is the fifth most frequently diagnosed cancer worldwide and the second most frequent cause of cancer death. The aim of this study is to identify the association between the expression of long non-coding RNA (lncRNA) MIR22HG and the clinical and tumor characteristics of patients with HCC, and to explore the prognostic significance of lncRNA MIR22HG on patients with HCC. We retrospectively reviewed 127 patients with HCC(42 female, 85 male) who were managed in our hospital between May 1^st^ 2010 and June 30^th^ 2016. The expressions of lncRNA MIR22HG were detected by real-time PCR. Prognostic factors were evaluated using Kaplan-Meier curves and Cox proportional hazards models. For the entire cohort of 127 patients, the normalized real-time PCR showed that the expression of lncRNA MIR22HG was lower in HCC tissues compared with corresponding nontumorous tissues. MTT assay showed that si-MIR22HG remarkably inhibited the proliferation tumor cells in three HCC cell lines including SMMC-7721, Huh-7 and Hep3B. Moreover, under-expression of MIR22HG was closely related to tumor encapsulation, microvascular invasion (MVI), and TNM stage. Cox proportional hazards analysis demonstrated that lncRNA MIR22HG under-expression was an independent risk factor associated with the prognosis of patients with HCC. In conclusion, we found that lncRNA MIR22HG expressed significantly lower in HCC tissues compared with non-tumorous tissues. Under-expression of lncRNAMIR22HG was an independent risk factor associated with the prognosis of patients with HCC.

## INTRODUCTION

Hepatocellular carcinoma (HCC) is the fifth most frequently diagnosed cancer worldwide and a sustaining high mortality [1], which was frequent in Asian countries and especially in China [2]. Many prog nostic factors of HCC have been identified, including serum alpha-fetoprotein (AFP) levels, tumor size, tumor multifocality, microvascular invasion, completeness of tumor removal and tumor metastases, etc [3, 4]. The the reason for the poor prognosis of patients with HCC is that only 30% to 40% of patients are diagnosed at the early stage which were candidates for potentially curative hepatectomy [5]. As a consequence, many patients have poor prognosis because the high rate of recurrence after hepatectomy or of intrahepatic metastases through invasion of portal or hepatic veins in the liver [6]. Therefore, exploring novelly diagnostic and prognostic factors is vital to facilitate screening of high risk patients and make decisions on the adjuvant therapy.

Long non-coding RNAs (lncRNAs) are transcribed RNA molecules with more than 200 nucleotides and can not code proteins [7]. Many studies have demonstrated the diverse cellular functions of lncRNAs including cell proliferation, cell differentiation, cell apoptosis and carcinogenesis [8-10]. Previous reports showed that lncRNAs played a essential role in HCC, while the mechanism was not clear and needed more elucidation [11]. Moreover, the lncRNA NR_028502.1, which is located in 17p13.3, a chromosomal region that is frequently deleted, hypermethylated, or shows loss of heterozygosity in liver cancer, was down-regulated in HCC [12, 13]. LncRNA NR_028502.1 has been identified as a lncRNA in The Encyclopedia of DNA Elements (ENCODE) project and was annotated as the human miR-22 host gene (MIR22HG). Previous reports showed that chromosome location and sequence similarity suggested that some lncRNAs might serve as the host genes of miRNAs and act in close association with miRNAs [14]. For example, lncRNA H19, a host gene of miR-675, generated mature miR-675-5p and miR-675-3p which were associated with tumor metastasis [15, 16]. However, the significance of lncRNA MIR22HG was not reported previously, and whether dysregulation of lncRNA MIR22HG associated with tumorigenesis of HCC is unknown.

With respect to HCC, few studies reported the association between lncRNA MIR22HG and the prognosis of patients with HCC after hepatectomy. In present study, we investigated the clinicopathological characteristics of patients with HCC to identify the association between lncRNA MIR22HG levels and the prognosis of HCC.

## RESULTS

### Patients’ characteristics

127 patients with HCC after curative resection were recruited into this study. The median follow-up was 4.2 years (range 3.7 months-9.6 years). The baseline characteristics of patients divided after MIR22HG detection were summarized in Table 1. Under-expression of MIR22HG was closely related to tumor encapsulation (P=0.006), microvascular invasion (MVI) (P=0.013), and TNM stage (P=0.020) (Table 1). While there is no significant relation with gender, age, HbsAg, HbeAg and tumor size etc (Table 1).

**Table 1:** Correlation between LncRNA MIR22HG expression and clinicopathologic features

Variables		LncRNA MIR22HG expression		
		Low(n=53)	High(n=74)	*P*
**Sex**	female	20	22	0.344
	male	33	52	
**Age**	median	53	51	0.251
	range	20-70	32-71	
**AFP level (μg/L)**	<400	25	32	0.661
	>400	28	42	
**HBsAg**	positive	42	59	0.947
	negative	11	15	
**HBeAg**	positive	36	47	0.606
	negative	17	27	
**Liver cirrhosis**	yes	26	43	0.313
	no	27	31	
**Edmondson-Steiner** **grade**	I-II	19	32	0.475
	III- IV	34	42	
**Diameter (cm)**	≤5	28	36	0.642
	>5	25	38	
**Encapsulation**	complete	15	39	0.006
	no	38	35	
**MVI**	yes	34	31	0.013
	no	19	43	
**Tumor number**	single	41	56	0.826
	multiple	12	18	
**TBL (umol/l)**	median	13.5	15.7	0.671
	range	4.26-68.32	4.27-72.41	
**Alb (g/dl)**	median	39.3	38.6	0.173
	range	24.5-51.4	23.6-53.8	
**ALT (U/L)**	median	63.2	67.3	0.801
	range	12.4-238.3	7.8-276.2	
PLT (^*^10^9^**/L)**	median	127	125	0.703
	range	28-375	21-382	
**INR**	median	1.05	1.04	0.285
	range	0.87-1.26	0.89-1.23	
**CR**	median	72	63	0.341
	range	40-147	38-172	
**TNM stage:**	I	6	19	0.020
	II	21	35	
	III	26	20	

AFP, alpha-fetoprotein; HBsAg, hepatitis B surface antigen; HBeAg, Hepatitis E antigen; MVI, microvascular invasion; TBIL, total bilirubin; ALB, albumin; ALT, alanine; PLT, platelet count; INR, international normalized ratio; CR, creatinine.

### Detection of MIR22HG in HCC tissues, corresponding nontumorous tissues and HCC cell lines

**Figure 1:**
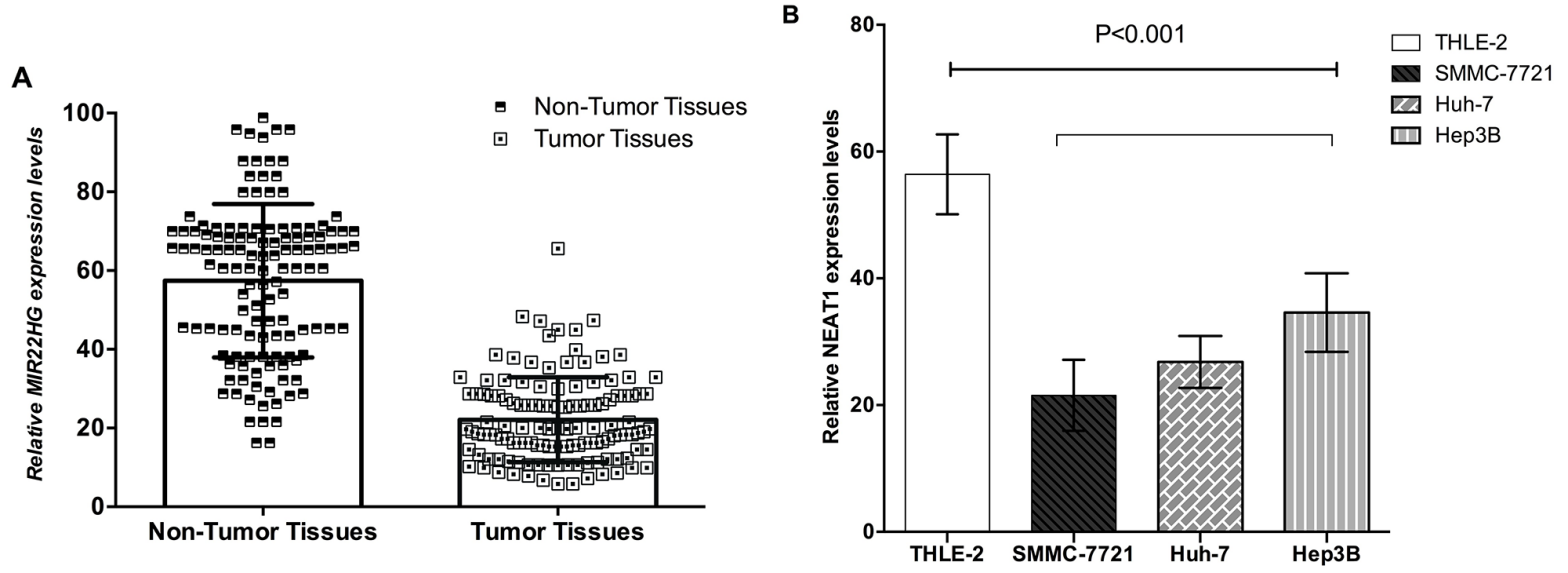
MIR22HG was down-regulated in HCC tissues and cell lines Relative MIR22HG concentration was detected using Real-Time qPCR. **(A)** MIR22HG expression levels were lower in HCC tissues than those in non-tumor tissues(p<0.001); **(B)** MIR22HG expression levels were lower in SMMC-7721, Huh-7 and Hep3B cell lines than the normal hepatic cell line (THLE-2) C(p<0.001).

MIR22HG expressed significantly lower in HCC tissues compared with non-tumorous tissues (P<0.001) (Figure 1A). MIR22HG was decreased obviously in the HCC cell lines including SMMC-7721 (P<0.001), Huh-7 (P<0.001) and Hep3B (P<0.001), compared with the control group of THLE-2 (Figure 1B).

### Over-expression of MIR22HG arrested cell proliferation

**Figure 2:**
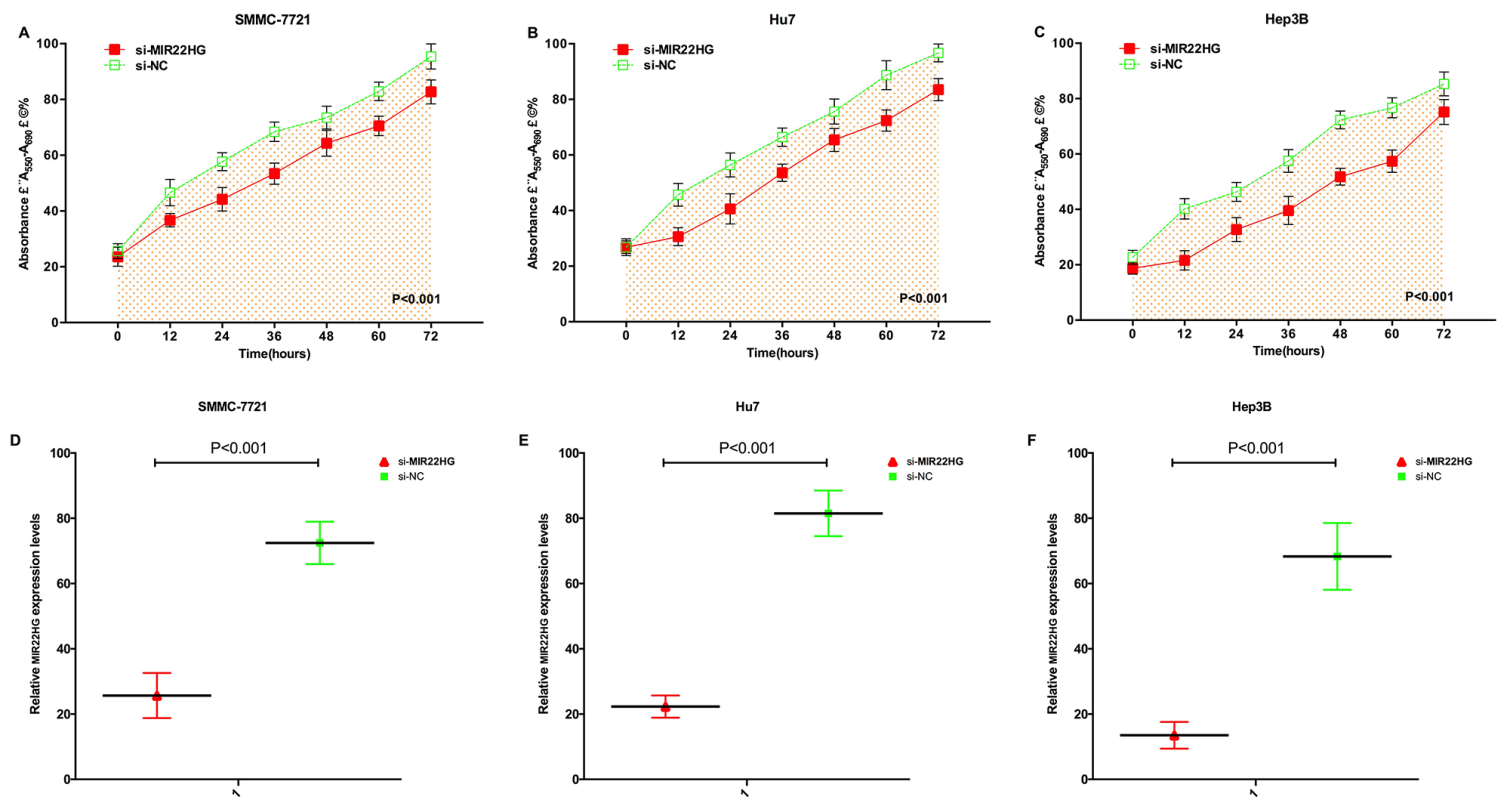
Expression changes of MIR22HG after transfection and over-expression of MIR22HG inhibited cell proliferation by MTT assay After transfection of si-MIR22HG or negative control si-NC, OD values were measured. ANOVA was used for the comparison of curves of cell proliferation. Cell proliferation inhibition was observed in HCC cell lines SMMC-7721 **(A)** Huh-7 **(B)** and Hep3B **(C)** cells (p<0.01). The si-MIR22HG was more significantly down-regulated in SMMC-7721 **(D)**, Huh-7 **(E)** and Hep3B **(F)** cells (p<0.01). Each experiment in three cell lines was performed in triplicate for three independent times.

MTT assay detected that si-MIR22HG remarkably inhibited the proliferation of three HCC cell lines (p < 0.001) (Figure 2A-2C). At 48 hours after transfection of si-MIR22HG and si-NC in SMMC-7721, Huh-7 and Hep3B cell lines, we detected the related MIR22HG expression level by qRT-PCR in HCC cell lines of SMMC-7721 (P<0.001), Huh-7 (P<0.001) and Hep3B (P<0.001) (Figure 2D-2F).

### Survival descriptions of different subgroups divided by MIR22HG expression

**Figure 3:**
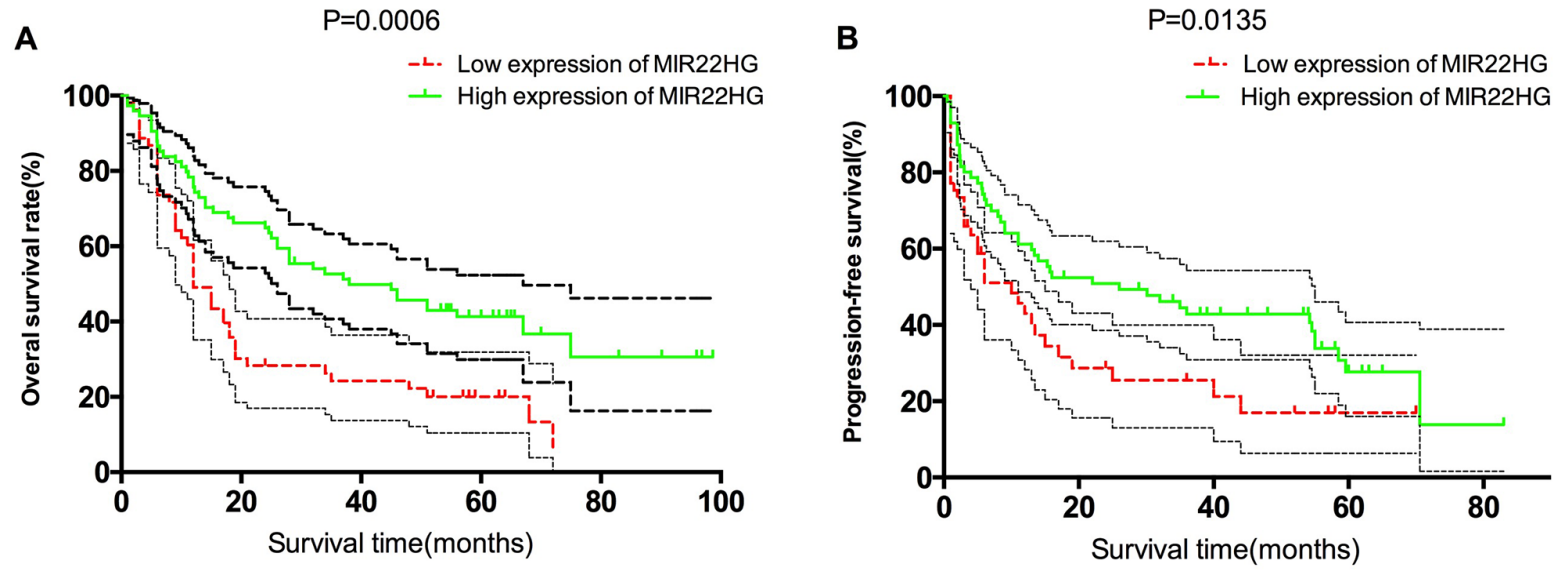
Overall survival and progressive-free survival estimates, **(A)** OS of patients stratified by MIR22HG expression levels (P = 0.0006); **(B)** PFS of patients stratified by MIR22HG expression levels (P = 0.0135).

Descriptive survival statistics and Kaplan-Meier curves suggested that under-expression of MIR22HG had prognostic significance in this relatively selected cohort. Under-expression of MIR22HG was associated with a decreasing 1-, 3-, 5-year OS rate from 79.7%, 52.6%, 41.3% to 60.4%, 26.3%, 20.2% (P=0.0006, Figure 3A). Meanwhile, under-expression of MIR22HG was associated with a decreasing 1-, 3-, 5-year PFS rate from 61.5%, 42.8%, 33.8% to 45.7%, 25.5%, 16.9%, respectively (P=0.0135, Figure 3B).

### Cox proportional hazard analysis

Cox proportional hazards models were then used to quantify the prognostic significance of risk factors after multivariable adjustment. Univariable Cox proportional hazards analysis demonstrated that TNM stage (P=0.003), tumor number (P=0.023), AFP>400 (ng/ml) (P=0.004), tumor encapsulation (P=0.017), microvascular invasion (P=0.013), tumor size (P<0.001) and under-expression of MIR22HG (P<0.001) were associated with a worse prognosis of patients with HCC. A multivariable analysis was performed after adjusting for competing risk factors. We identified that TNM stage (P=0.016), tumor number (P=0.012), AFP>400 (ng/ml) (P=0.008), MVI (P=0.024), tumor size (P<0.001) and under-expression of MIR22HG (P=0.021) were significant prognostic factors associated with HCC patients (Table 2).

**Table 2:** Cox proportional hazard regression analyses

Variable	Univariate			Multivariate		
	HR	95%CI	P value	HR	95%CI	P value
**Age in yr (median range)**	0.983	0.782-1.291	0.371			
**Gender, male:female**	1.072	0.842-1.206	0.618			
**HbsAg: positive: negative**	1.156	0.901-1.405	0.251			
**HBeAg: positive: negative**	1.174	0.916-1.641	0.207			
**Liver cirrhosis: with: witout**	1.215	0.823-1.629	0.103			
**Antiviral therapy**	1.152	0.891-1.603	0.258			
**TBL (umol/l): >17:≤17**	0.803	0.606-1.281	0.352			
**ALB (g/dl): >40: ≤40**	0.961	0.759-1.319	0.184			
**ALT (U/L): >40: ≤40**	1.031	0.983-1.017	0.439			
**TNM stage: I:II:III**	1.593	1.222-3.268	0.003	1.621	1.286-2.723	0.016
**No. tumor: Solitary :Multiple**	1.856	1.234-3.136	0.023	1.526	1.351-2.602	0.012
**AFP(≤400ug/L *vs* >400ug)**	1.764	1.372-3.721	0.004	1.432	1.230-2.721	0.008
**Edmondson-Steiner grade: I+II:III**	1.088	0.827-1.430	0.548			
**Tumor encapsulation: No:Complete**	1.411	1.064-1.871	0.017	1.122	0.843-1.493	0.435
**Micro-vascular invasion(+/-)**	1.521	1.378-2.762	0.013	1.492	1.235-2.743	0.024
**Tumor size (≤5 cm *vs* >5 cm)**	1.443	1.193- 4.351	<0.001	2.539	1.520-4.256	<0.001
**under-expression of LncRNA MIR22HG**	1.957	1.444- 2.621	<0.001	1.05	1.073-1.892	0.021

CI, indicates confidence; TBL, total bilirubin; ALB, albumin; ALT, alanine aminotransferase; PT, prothrombin time; PLT, blood platelet; AFP, alpha-fetoprotein.

## DISCUSSION

In the past decade, HCC incidence is increasing because of the rising incidence in Western Europe and Northern America [20, 21]. The outcomes for patients with HCC have improved markedly over the last 30 years due to the presence of various treatments and advances in surgical treatment [22].

In recent years, the number of articles focused on lncRNAs has increased greatly. Recent studies have demonstrated that certain lncRNAs are specifically correlated with certain classes of cancer and the different expression level of lncRNAs may function as an indicator for metastasis and prognosis [23-25]. It described lncRNAs as RNA molecules might have the function as primary or spliced transcripts. Given the large-scale regulation of lncRNAs in HCC, it is highly possible that lncRNAs are directly linked to the development of hepatocellular carcinoma. Abnormally expressed lncRNAs are found to play a key role in liver cancer and metastasis and prognosis [26].

More and more research has focused on the contribution of lncRNAs in the development of liver cancer. Notably, mutation of the MIR22HG in the miR-22-3p region demonstrated that the mutant MIR22HG retained the abilities to inhibit HCC cell proliferation, migration, and invasion. This indicated that the function of MIR22HG was not totally dependent on the derived miR-22-3p. Investigation of the molecular mechanisms by which MIR22HG contributes to tumor suppression identified the involvement of HuR, which is known to regulate the splicing, stability, or translation of thousands of both coding and non-coding RNAs [27, 28].

In this study, we presented strong evidence that lncRNA MIR22HG expressed significantly lower in HCC tissues compared with non-tumorous tissues and lncRNA MIR22HG was decreased obviously in the HCC cell lines including SMMC-7721, Huh-7 and Hep3B. MTT assay detected that si-MIR22HG remarkably inhibited the proliferation of three HCC cell lines. Moreover, under-expression of MIR22HG was correlated with tumor progression and was found to be an independent predictor for the prognosis of patients with HCC after curative resection.

However, there are limitations of this study: (1) the sample size is too small in this study, and further larger sample study is needed to confirm the present experimental results. (2) whether under-expression of MIR22HG have the optimal specificity and sensitivity for liver cancer diagnosis also needs future confirmation.

In conclusion, we found that lncRNA MIR22HG expressed significantly lower in HCC tissues compared with non-tumorous tissues. Under-expression of lncRNAMIR22HG was an independent risk factor associated with the prognosis of patients with HCC.

## MATERIALS AND METHODS

### Patients and tissue samples

A total of 127 patients with primary HCC who underwent a curative liver resection at the 302 Hospital of People’s Liberation Army, were included in this retrospective study. These patients were diagnosed as HCC between May 1^st^ 2010 and June 30^th^ 2016. The tissues of HCC were immediately frozen in liquid nitrogen after surgical removal and stored at -80°C until use. HCC diagnosis was based on WHO criteria. Tumor staging was determined according to the sixth edition of the tumor-node-metastasis (TNM) classification of the International Union against Cancer. The characteristics of patients were shown in Table 1. The study was approved by the Research Ethics Committee of 302 Hospital of People’s Liberation Army. Informed consent was obtained from all patients.

### Cell lines and culture conditions

Human HCC cell lines (SMMC-7721, Huh-7 and Hep3B cells) were purchased from the Institute of Cell Biology, Chinese Academy of Sciences (Shanghai, China). SMMC-7721, Huh-7 and Hep3B cell lines were cultured in RPMI-1640 Medium (Invitrogen, Carlsbad, CA, USA) supplemented with 10% fetal bovine serum (FBS) and 1% penicillin-streptomycin. The THLE-2 cells were cultured in BEGM (Bronchial Epithelial Medium, In virtogen, Carlsbad, CA, USA) supplemented with a mixture of 0.01 mg/ml fibronectin, 0.03 mg/ml bovine collagen type I and 0.01 mg/ml bovine serum albumin dissolved in BEBM medium.

### MTT assay

The HCC cells proliferation was also measured by using 3-4, 5-dimethylthiazol-2-yl-2, 5-diphenyl-tetrazolium bromide (MTT) assay. Cells were grown in a 96-well plate for 24 hours, transfected with si-MIR22HG or negative control si-NC and incubated in normal medium. Cells were seeded in 0.1 mg/ml MTT for 4 hours and lysed in dimethyl sulfoxide (DMSO) at room temperature for 10 minutes. The absorbance in each well was detected by a microplate reader (Bio-Rad, Hercules, CA, USA) at 0, 12, 24, 36, 48, 60 and 72h after transfection.

### SiRNA transfection

Cell Transfection GC cell lines were transfected with siRNA using Lipofectamine 2000 (Invitrogen, Carlsbad, CA, USA), according to the manufacture’sprotocol. MIR22HG-specic siRNAs (si-MIR22HG): Sense GAUUGAUGGA GGGUGUUGGA and antisense UUCUUCACUUCCAUCCCAUC and negative control siRNA (si-NC) were purchased from GenePharma, Shanghai, China.

### Real-time quantitative PCR

We typically extracted 2 μg to 9 μg of total RNA, and OD260/280 ratios typically ranged from 1.8 to 2.0, indicating high RNA purity. 10 ng of total RNA was used for each miRNA quantification. miRNA detection was performed run on the Eppendorf Mastercycler EP Gradient S (Eppendorf, Germany) using commercial assays (TaqMan microRNA assays; Applied Biosystems, Foster City, CA, USA) for miRNAs. Relative quantification was calculated using 2^-ΔΔCt^, where Ct is cycle threshold. Normalization was performed with universal small nuclear RNA U6 (RNU6B). Each sample was examined in triplicate, and the mean values were calculated. mRNA levels in tumor samples/nontumorous samples of 0.5-fold was defined as under-expression of the gene, whereas a ratio of 2.0-fold was defined as over-expression.

### Follow-up

Postoperative serum AFP and abdominal ultrasound were carried out in all patients monthly. Patients received abdominal contrast-enhanced CT scan or MRI once every 3 months in the first two years after surgery, and once every 6 months thereafter. Further investigations were carried out when clinically indicated or when tumor recurrence was suspected. Outcome definitions: Complete resection was defined as resection of all tumor sites on the basis of surgical findings and postsurgical images. Overall survival (OS) was defined as the period from the date of first treatment until death. Patients who did not experience an event were censored on the date of last contact. Progressive free survival (PFS) was defined as the period from the date of first treatment until an occurrence of an event (progressive disease, death, diagnosis of a second malignant neoplasm), whichever occurred first.

### Statistical methods

Continuous variables were expressed as mean ± SD (standard deviation) and compared using a two-tailed unpaired Student’s t test; categorical variables were compared using χ2 or Fisher analysis. The cut-off of AFP level was defined by the receiver-operating characteristic (ROC) curve analysis [17]. Life-table estimates of survival time were calculated according to the Kaplan and Meier methodology [18]. The Greenwood formula was used for the standard deviation. A Cox proportional hazards regression approach [19] was chosen for the evaluation of PFS and OS as the primary end-point. Potential prognostic variables were analyzed both univariately with one factor taken at a time, and then in a multivariate model combining all factors. Results were showed as hazard ratios (HR) and their 95% confidence intervals (CI) A HR > 1 indicated an elevated risk with respect to the reference category. A confidence interval which did not include the value 1 indicated statistical significance at the 5% level. It should be noted that this was a retrospective evaluation and therefore statistical significance should be interpreted with caution. All statistical evaluations will be carried out using SPSS software (Statistical Package for the Social Science, version 15.0, SPSS Inc, Chicago, IL) and GraphPad Prism 5 (Version 5.01, GraphPad Software, Inc., USA).
